# Acute Truncal Lymphedema Secondary to Axillary Metastatic Melanoma Presenting Like Cellulitis

**DOI:** 10.1155/2017/5462929

**Published:** 2017-01-15

**Authors:** Shelley J. E. Hwang, Benjamin Y. Kong, Shaun Chou, Deepal Wakade, Matteo S. Carlino, Pablo Fernandez-Penas

**Affiliations:** ^1^Department of Dermatology, Westmead Hospital, Westmead, NSW, Australia; ^2^Sydney Medical School, The University of Sydney, Sydney, NSW, Australia; ^3^Crown Princess Mary Cancer Centre, Westmead Hospital, Westmead, NSW, Australia; ^4^Department of Tissue Pathology, Westmead Hospital, Westmead, NSW, Australia; ^5^Melanoma Institute Australia, Sydney, NSW, Australia

## Abstract

There are reported cases of diphencyprone used in treating cutaneous metastases of melanoma. Here, we report a patient with previous primary melanoma on his left back treated with surgical excision and lymphadenectomy, followed by radiotherapy for the recurrent tumor on the primary site. Despite radiotherapy and treatment with dabrafenib and trametinib, in-transit metastases have developed and topical diphencyprone was applied to these metastases. Six weeks later, the patient developed fever and a spreading erythematous tender indurated plaque covering the left side of the body including axillae, back, and flank, clinically suggestive of cellulitis. Systemic antibiotic therapy did not improve the condition and a biopsy showed sparse lymphocytic infiltrate. With the diagnosis of possible acute lymphedema, a CT scan was requested that showed significant axillary lymph node metastasis. The fever was considered secondary to dabrafenib and trametinib therapy. This case highlights that, in patients with lymphadenectomy, atypical forms of lymphedema on the body may appear. Truncal lymphedema is an infrequent event.

## 1. Manuscript

Upper limb lymphedema commonly complicates the detection and management of axillary nodal metastasis from melanoma, and its prevalence after radical axillary dissection for melanoma is reported to vary from 1 to 12% [1]. However, lymphedema affecting the trunk is rarely described; hence considering this as a differential diagnosis would be unusual.

A 68-year-old Caucasian male presented with rapidly progressing local recurrence and in-transit deposits of cutaneous melanoma on his left upper back. A primary BRAF*V600E* positive 0.9 mm Breslow thickness superficial spreading malignant melanoma was treated with wide local excision the previous year, which recurred within five months and he was found to have in-transit metastasis with left axillary palpable lymphadenopathy. He subsequently received a level I–III axillary dissection (10 of 34 lymph nodes were positive for melanoma) together with an adjuvant high dose radiotherapy (55 Gy in 20 fractions). Despite locoregional radiotherapy, cutaneous recurrences appeared within the irradiated field of his primary site. Subsequently, the patient was commenced on the combination of dabrafenib and trametinib. Four months later, topical immunotherapy with diphencyprone (DPCP) was commenced as an adjunct therapy [2–5] to the locoregional metastasis. Concurrently, the patient was initiated on low dose oral prednisone (20 mg daily) to control fever from dabrafenib and trametinib.

After four applications of DPCP, the patient developed mild erythema around the cutaneous metastasis but not on the melanoma deposits. Betamethasone valerate cream was applied to the surrounding skin, and as there were no signs of adequate reaction achieved on the lesions, the frequency of DPCP application was increased. Dabrafenib and trametinib were ceased due to worsening systemic adverse events (fever, nausea, and vomiting).

**Figure 1:**
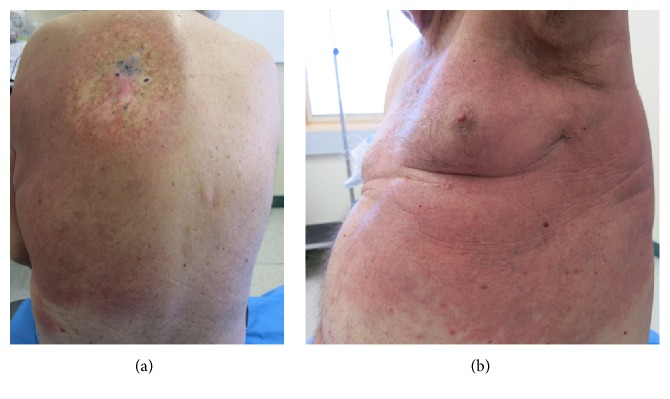
(a) Erythematous cutaneous reactions on left upper back spreading to lower back and to (b) axilla and flank.

Twelve days later, the patient presented with extensively spread erythematous reaction on his left upper and lower back (Figure 1(a)), axilla, flank, and abdomen (Figure 1(b)). The reaction was limited to midline of his back. He had fever and elevated white cell count. The differential diagnosis of allergic contact dermatitis and cellulitis was considered. A skin biopsy was taken and empirical oral flucloxacillin was commenced whilst DPCP was ceased.

Two days later, due to worsening cutaneous reaction and persistent fever, he was hospitalized to receive intravenous flucloxacillin. Repeated blood cultures were negative. A skin biopsy showed only mild psoriasiform hyperplasia, occasional dermal eosinophils, and no neutrophils. The patient was commenced on a weaning dose of oral prednisone to reduce inflammation.

Two weeks later, the skin became sclerodermatoid and an incisional skin biopsy showed focal parakeratosis, mild superficial perivascular chronic inflammation, patchy papillary dermal edema, and vascular ectasia. There was no spongiosis or significant acute inflammation. A consensus of acute lymphedema secondary to potentially failed lymphatic drainage from underlying recurrence of the disease was made. Computed tomography (CT) scans of the chest, abdomen, and pelvis confirmed recurrence of tumor at left axillae. Subsequently, the patient was commenced on pembrolizumab. Unfortunately, the patient deteriorated and passed away in four weeks.

Limb lymphedema is a common complication related to radical lymph nodes clearance [1]. However, lymphedema affecting the trunk is substantially less common [6]. It has been reported that truncal lymphedema involving chest, axilla, shoulder, breast, and back regions can appear in some breast cancer survivors [7, 8]. As high as 48% patients with breast cancer who had undergone axillary dissection with positive nodes are reported to develop breast edema a year after the surgery [9]. However, there is a lack of literature describing truncal lymphedema appearing in patients with advanced melanoma.

Our case illustrates a cellulitis-like clinical presentation on the trunk that masked acute lymphedema from secondary axillary metastasis. This is more relevant in patients with advanced melanoma who have progressed whilst on targeted therapy [10]. In our case, the differential diagnoses were difficult to ascertain as treatment with DPCP could trigger acute contact eczema with a similar clinical presentation. Although the activity of DPCP could have been a factor in the development of this atypical form of lymphedema, the absence of inflammation in the biopsies does not support it.

Our case highlights that, in patients with lymphadenectomy for melanoma, atypical forms of lymphedema on the body may appear. Truncal swelling mimicking cellulitis may be an early clinical sign of underlying tumor progression. However, being an infrequent event, it may be challenging for clinicians to reach this diagnosis early.

## Abbreviations

DPCP: Diphencyprone
CT: Computed tomography.
